# Locked Out: Phoenixin-14 Does Not Cross a Stem-Cell-Derived Blood–Brain Barrier Model

**DOI:** 10.3390/brainsci13070980

**Published:** 2023-06-22

**Authors:** Martha A. Schalla, Sabrina Oerter, Alevtina Cubukova, Marco Metzger, Antje Appelt-Menzel, Andreas Stengel

**Affiliations:** 1 Charité Center for Internal Medicine and Dermatology, Department for Psychosomatic Medicine; Charite—Universitätsmedizin BerlinCorporate Member of Freie Universität Berlin, Humboldt-Universität zu Berlin and Berlin Institute of Health, 12203 Berlin, Germany; 2 Department of Gynecology and Obstetrics, HELIOS Kliniken GmbH, 78628 Rottweil, Germany; 3 Department of Psychosomatic Medicine and Psychotherapy, University Hospital Tübingen, Osianderstr. 5, 72076 Tübingen, Germany; 4 Fraunhofer Institute for Silicate Research ISC, Translational Center Regenerative Therapies (TLC-RT), 97070 Würzburg, Germany; 5 Chair Tissue Engineering and Regenerative Medicine (TERM), University Hospital Würzburg, 97070 Würzburg, Germany

**Keywords:** appetite regulation, blood–brain barrier, brain–gut axis, gastrointestinal tract, hypothalamus, in vitro techniques, induced pluripotent stem cells, peptides, phoenixin-14

## Abstract

Phoenixin-14 is a recently discovered peptide regulating appetite. Interestingly, it is expressed in the gastrointestinal tract; however, its supposed receptor, GPR173, is predominantly found in hypothalamic areas. To date, it is unknown how peripherally secreted phoenixin-14 is able to reach its centrally located receptor. To investigate whether phoenixin is able to pass the blood–brain barrier, we used an in vitro mono-culture blood–brain barrier (BBB) model consisting of brain capillary-like endothelial cells derived from human induced-pluripotent stem cells (hiPSC-BCECs). The passage of 1 nMol and 10 nMol of phoenixin-14 via the mono-culture was measured after 30, 60, 90, 120, 150, 180, 210, and 240 min using a commercial ELISA kit. The permeability coefficients (PC) of 1 nMol and 10 nMol phoenixin-14 were 0.021 ± 0.003 and 0.044 ± 0.013 µm/min, respectively. In comparison with the PC of solutes known to cross the BBB in vivo, those of phoenixin-14 in both concentrations are very low. Here, we show that phoenixin-14 alone is not able to cross the BBB, suggesting that the effects of peripherally secreted phoenixin-14 depend on a co-transport mechanism at the BBB in vivo. The mechanisms responsible for phoenixin-14′s orexigenic property along the gut–brain axis warrant further research.

## 1. Introduction

Food intake regulation is a crucial physiological function of the body that is essential for survival [1]. The most significant physiological systems involved in this regulation include the hypothalamus and the brainstem, the food intake-regulating centers of the central nervous system, as well as the gastrointestinal tract, responsible for nutrient sensing and absorption [2]. These two communicate via the bidirectional gut–brain axis, which consists of signaling across the autonomic nervous system, including the vagal nerve, but additionally, there is growing evidence for the crucial role of peptides along this axis [3].

Phoenixin is a highly conserved peptide that is expressed in various species such as humans, rodents, pigs, cows, chicken, and fish [4]. It is found in different amino acid lengths, with phoenixin-14 and phoenixin-20 being the most prevalent forms [4]. Its expression is widespread, including central and peripheral tissues [4]. The peripheral organs shown to express phoenixin listed in order of decreasing concentration are the heart, thymus, esophagus, stomach, spleen, pancreas, lung, kidney, jejunum, duodenum, ileum, and colon [4]. Findings of phoenixin expression in the gastrointestinal tract were corroborated by immunohistological examinations showing phoenixin immunoreactivity in crypts of the duodenum, jejunum and ileum, and outer endocrine islets of the pancreas [5]. Interestingly, phoenixin mRNA expression, e.g., in the brain, liver, muscle, and gonads of fish were shown to be affected by fasting and refeeding [6,7]. Furthermore, in humans, circulating phoenixin levels correlated positively with body weight [8], indicating a role of phoenixin-14 in appetite regulation.

Indeed, phoenixin was shown to increase light phase food intake in rats after intracerebroventricular injection [4], indicating the centrally mediated appetite-stimulating effect of phoenixn-14, supposedly via the brainstem and hypothalamic centers involved in food intake. This suggestion was corroborated by observations of stimulated firing frequency and depolarization of the nucleus of the solitary tract (NTS) as well as [9] of magnocellular neurons in the paraventricular nucleus [10] by phoenixin-14. The NTS is the regulating center of the hindbrain where satiety signals are processed to promote the termination of a meal, while the PVN receives its input in form of agouti-related protein (AGRP) and neuropeptide Y (NPY) from pro-opiomelanocortin (POMC) neurons [1,11,12], which are the main mediators involved in the central homeostatic regulation of feeding [13]. The dependence of phoenixin-14′s orexigenic effect on these central circuits is also shown by the absence of phoenixin-14-induced hyperphagia due to NPY receptor 1 and 5 inhibition [14], receptors which are expressed on the PVN [11]. Notably, since the onset of phoenixin-14′s orexigenic effect in rats was delayed by two hours, phoenixin-14 signaling is likely associated with the recruitment of additional downstream mediators. This is also indicated by the significant overlap in the expression of phoenixin with another food intake-regulating peptide, nesfatin-1 [15]. Centrally applied phoenixin-14 activates nesfatin-1 immunoreactive neurons in the brain [16], and nesfatin-1 itself is highly co-expressed with POMC/cocaine and amphetamine-regulated transcript (CART) and NPY [15,17]; thus, it could be a modulator of phoenixin-14′s orexigenic effects.

In addition to phoenixin-14′s effect on food intake, it was also shown that this peptide is able to affect various other physiological functions including reproduction and memory and stress reactions, which were demonstrated to be predominantly centrally mediated as well [18]. Early on, it was suggested that phoenixin’s effects are mediated via a G-protein coupled receptor (GRP) based on observations of disrupted phoenixin-induced luteinizing hormone secretion by GRP173 blockage [10,19,20]. In vitro, phoenixin-induced GPR173 signaling activated the cAMP/PKA pathway of CREB to induce mRNA expression in GnRH neurons [19], vasopressin secretion into circulation [10], memory recognition [21], and anxiolytic effects [22]. GRP173 is identified in various organs: its mRNA is highly expressed among others in the PVN, supraoptic nucleus (SON), and ventromedial hypothalamus (VMH), and is found in moderate density in the arcuate nucleus (ARC) and lateral hypothalamic area [20].

The observations that phoenixin is expressed in various sections of the gastrointestinal tract [4], increases food intake after central application [23], and its supposed receptor in food intake-regulating hypothalamic regions such as PVN and ARC [20] point towards a significant role of phoenixin along the gut–brain axis. This is further supported by findings showing that pre- and postprandial levels of phoenixin in the plasma differ significantly [6,7,24]. There are two mains ways, which are currently discussed, that peptides secreted from the gastrointestinal tract exert their hypothalamic actions: either via afferent fibers of the vagal nerve or by crossing the blood–brain barrier [12]. Thus, this study aimed at investigating the capacity of phoenixin-14 to directly cross the blood–brain barrier, using an in vitro model.

## 2. Materials and Methods

To examine whether phoenixin-14 is able to cross the blood–brain barrier, we used commercially purchased phoenixin-14 in two different concentrations based on previously applied doses in vivo (since 100 nMol of phoenixin-14 is a supraphysiological concentration, it was used only in testing for cytotoxicity and not for the transport assay) [23]. The applied human induced-pluripotent stem-cell-derived BBB in vitro model was characterized by a high correlation to the human in vivo situation [25,26,27,28,29,30]. Finally, to measure the concentration of phoenixin-14 in the cell culture, a commercially purchased ELISA was used as previously described [31].

### 2.1. Cultivation of a Single-Cell Blood–Brain Barrier Model

Human induced-pluripotent stem cells (hiPSC; IMR90-4, WiCell Research Institute, Madison, WI, USA) were cultured in mTeSR™1 medium (StemCell Technologies, Vancouver, BC, Canada). The hiPSC-derived brain capillary-like endothelial cell (hiPS-BCEC) BBB model was built as previously described [29,30,32].

In brief, using Accutase^®^ solution (Sigma-Aldrich, Saint Louis, MO, USA), hiPSCs were dissociated into single cells and seeded in mTeSR™1 medium (StemCell Technologies Inc.) including 10 M Y-27632 dihydrochloride (Tocris, Bristol, UK) on Matrigel^®^ Growth Factor Reduced (GFR) Basement Membrane Matrix, Phenol Red-free (Corning, Corning, NY, USA) coated 6-well plates at a density of 7.5 × 103 hiPSC/cm^2^. The medium was changed daily until the cells reached a density of 2.5–3.5 × 104 hiPSC/cm^2^. To induce endothelial and neural cell co-differentiation, the medium was changed to an unconditioned medium (UM) consisting of DMEM/F-12 containing 20% KnockOut™ serum replacement (Thermo Fisher Scientific, Waltham, MA, USA), 1% MEM NEAA (Thermo Fisher Scientific), 1 mM L-glutamine (Sigma-Aldrich), and 0.1 mM β-Mercaptoethanol (Thermo Fisher Scientific), with daily medium changes for 6 days. For the next two days, cells were treated with EC++ medium, consisting of human endothelial-SFM (hESFM, Thermo Fisher Scientific) supplemented with 200 × diluted serum-free B-27™ supplement (Thermo Fisher Scientific), 20 ng/mL hbFGF (PeproTech, Cranbury, NJ, USA), and 10 μM all-trans RA (Sigma-Aldrich). No medium change occurred on day 7. Using Accutase^®^, cells were seeded in EC++ medium at a density of 1 × 106 hiPSC/cm^2^ onto 24-well ThinCert^®^ cell culture inserts, with a pore size of 0.4 µm (Greiner BioOne, Frickenhausen, Germany), coated with 400 μg/mL collagen IV (Sigma-Aldrich) and supplemented with 100 μg/mL fibronectin (Thermo Fisher Scientific). Cells were cultured in EC++ medium for 24 h, followed by treatment with EC medium (hESFM + B27, w/o hbFGF and RA) for another 24 h, yielding purified hiPS-BCECs.

To verify human BBB-like characteristics prior to transport studies, transendothelial electrical resistances (TEERs) were determined in the empty hiPSC-BCECs models with a Millicell ERS-2 voltohmmeter (Merck Millipore, Billerica, MA, USA) equipped with a STX3 electrode (World Precision Instruments Germany GmbH, Friedberg, Germany) as described before [12]. Briefly, TEER measurements were performed 40 min after medium change. Each model was measured at three positions, monitoring and excluding a possible data drift due to temperature fluctuations, and duplicates were used for each experiment in three independent biological replicates. To yield the TEER values [Ω*cm^2^] resulting from hiPSC-derived BCECs, average TEERs of empty inserts coated with collagen IV/fibronectin were subtracted and values were multiplied by insert surface.

### 2.2. Toxicity Studies

Cytotoxicity of phoenixin-14 against hiPS-BCECs was evaluated for three concentrations (1, 10, and 100 nMol) after 4 h incubation with untreated (negative control) and 1% Sodium Dodecyl Sulfate (SDS, Carl Roth, Karlsruhe, Germany)-treated (positive control) hiPS-BCECs as controls. After treatment, a CellTiter-Glo^®^ 2.0 Cell Viability Assay (Promega, Mannheim, Germany) was performed according to the manufacturer’s instructions. Luminescence was measured with an Infinite M200 fluorescence reader (Tecan Group, Männedorf, Switzerland).

### 2.3. Transport Studies

Transport assays were performed in cell culture inserts (24-well format) on an orbital shaker (Edmund Buhler GmbH, Bodelshausen, Germany) at 100 rpm, 37 °C, 95% humidity, and 5% CO_2_. The concentrations of 1 and 10 nMol phoenixin-14 for the transport studies were chosen based on previous data showing that intracerebroventricularly applied phoenixin-14 at a concentration of 1.7 nMol significantly affected food intake in vivo [23]. Stock solutions of commercially purchased phoenixin-14 (079-01, Phoenix Pharmaceuticals, Inc., Karlsruhe, Germany,) were diluted with EC medium to the desired concentrations (1 nMol or 10 nMol). In total, 200 µL of the final concentrations of phoenixin-14 were applied to the apical side of the BBB model. The basolateral side was supplied with 800 μL of EC medium.

The transport was evaluated with a total incubation time of 4 h. Half-hourly, the basolateral medium was collected and replaced with fresh EC medium. As a control, the substances were incubated on empty collagen IV-/fibronectin-coated inserts to exclude major phoenixin-14 adsorption on the membrane. Data from our previously published hiPS-BCEC-based transport studies of caffeine, loratadine, and FITC-dextran 40 kDa [32] as control substances were used for comparison.

### 2.4. ELISA

The concentration of phoenixin-14 in the apical and basolateral compartment was measured using a commercially purchased ELISA Kit (EK-079-01, Phoenix Pharmaceuticals). The intra-assay variability was 9.7%. The sensitivity for phoenixin was 0.07 ng/mL, while the linear detection ranged from 0.36 to 3.26 ng/mL (Phoenix Pharmaceuticals).

### 2.5. Statistical Analysis

Cytotoxicity studies were performed in triplicate and three independent biological replicates. Transport studies were performed in duplicate and repeated three times. Statistical analysis was conducted with SPSS 28 (IBM Corp. 2021, IBM SPSS Statistics for Windows, Version 28.0, Armonk, NY, USA). When feasible based on the number of values, data were tested for normality using the Kolmogorov–Smirnov test; otherwise, they were assumed to be non-normally distributed. Results from the toxicity studies were analyzed using Kruskal–Wallis test and significance values were adjusted according to Bonferroni correction regarding multiple testing. To compare the results of the transport studies, an unpaired *t*-test was applied. Data are presented as mean ± SEM. The level of significance was defined as *p* < 0.05 (*).

## 3. Results

### 3.1. TEER Values

**Figure 1:**
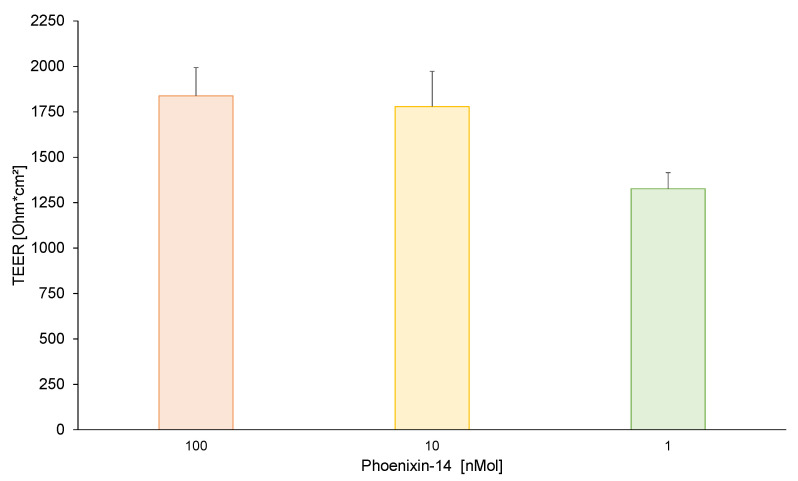
Using a Millicell ERS-2 voltohmmeter equipped with a STX3 electrode transendothelial electrical resistance (TEER) in the empty hiPSC-BCEC model prior to testing with 1, 10, and 100 nMol of phoenixin-14 were assessed. No significant differences between the three concentrations were found. All data are expressed as mean ± SEM.

TEER values in the untreated hiPSC-BCEC model of 1, 10, and 100 nMol of phoenixin-14 prior to the transport study were 1325.85 ± 89.57, 1778.41 ± 194.29, and 1837.63 ± 155.34 Ω*cm^2^, respectively, and not significantly different between each other (*p* > 0.05, Figure 1). The mean standard deviation of the individual TEER values for 1, 10, and 100 nMol of phoenixin-14 was 27.84, 12.35, and 33.61 Ω*cm^2^, respectively (*p* > 0.05).

### 3.2. Cytotoxicity Studies

**Figure 2:**
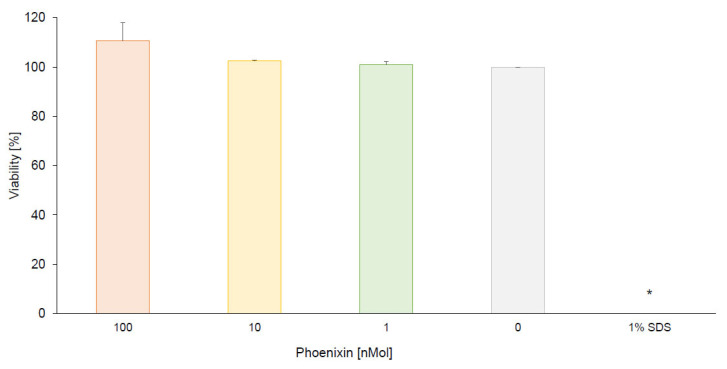
Cell viability of hiPSC-BCECs treated with phoenixin-14 was determined in comparison with an untreated control. Using CellTiter-Glo^®^ 2.0 Cell Viability Assay, viability of hiPSC-BCECs after incubation of 100, 10, and 1 nMol of phoenixin and 1% SDS was assessed. All data are expressed as mean ± SEM. * *p* < 0.05 vs. 100 nMol phoenixin-14.

Cytotoxicity of phoenixin-14 against hiPSC-BCECs was evaluated after 4 h incubation. The cell viability of treated hiPSC-BCECs was determined in comparison to untreated hiPS-BCECs (negative control). After incubation with phoenixin-14 in a concentration of 1, 10, and 100 nMol, viability was 101.05 ± 0.73%, 102.61 ± 0.07%, and 110.64 ± 4.23 (*p* > 0.05 compared with untreated hiPSC-BCECs, Figure 2), respectively. Treatment with 1% SDS reduced hiPSC-BCEC viability to 0.37 ± 0.02% (*p* < 0.05 compared with 100 nMol phoenixin-14, Figure 2).

### 3.3. Transport Assays

**Figure 3:**
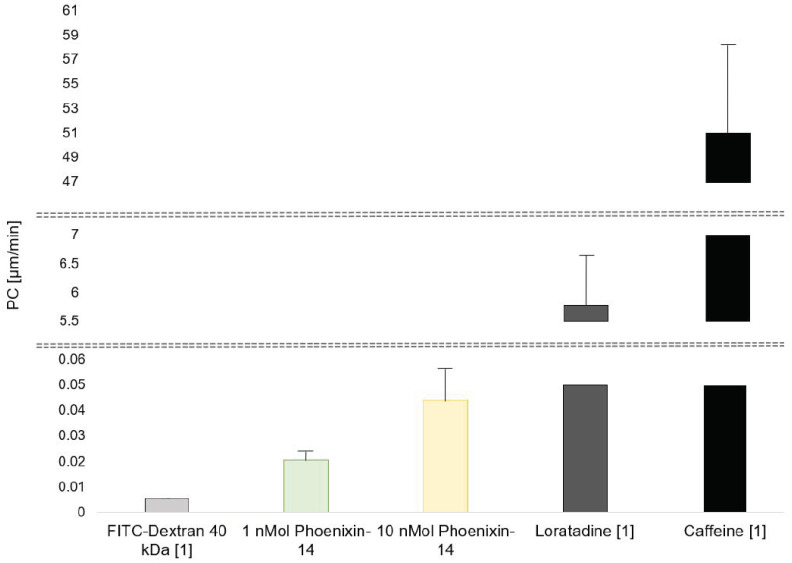
Using an hiPSC-derived blood–brain barrier model, the permeability coefficient (PC) of 1 and 10 nMol of phoenixin-15 was assessed. No significant difference between 1 and 10 nMol of phoenixin-14 was observed. In comparison, PCs of FICT-dextran 40 kDa (known to not cross the BBB), loratadine, and caffeine (both known to cross the BBB) based on previously published data are also shown [32]. All data are expressed as mean ± SEM.

In three independent experiments, the permeability coefficients (PC) of 1 nMol of phoenixin-14 were 0.014, 0.028, and 0.021 µm/min. Taken together, the PC of 1 nMol of phoenixin-14 was 0.021 ± 0.003 µm/min, resulting in clearance value of 0.683 × 10^−6^ ± 0.139 × 10^−6^ mL/min. The permeability coefficients of 10 nMol of phoenixin-14 were 0.059, 0.056, and 0.019 µm/min in each experiment. Thus, 10 nMol of phoenixin had a PC of 0.044 ± 0.013 µm/min. Based on this PC, the clearance value was 1.779 × 10^−6^ ± 0.817 × 10^−6^ mL/min. The PCs of 1 nMol and 10 nMol of phoenixin-14 were not significantly different (*p* = 0.085; Figure 3). In comparison, based on our previously published experiments in the hiPSC-based BBB model, caffeine had a PC of 51.06 ± 9.90 µm/min, loratadine’s PC was 5.78 ± 0.83 µm/min, and the PC of FITC-dextran 40 kDa was 0.0054 ± 0.0007 µm/min [32] (Figure 3).

As demonstrated by the very low PC values of 0.021 ± 0.003 and 0.044 ± 0.013 µm/min of 1 nMol and 10 nMol of phoenixin-14, respectively, phoenixin-14, independently of its concentration, passed the hiPSC-derived blood barrier model only in very insignificant amounts. These values are comparable to solutes such as FITC-Dextran 40, which are known to be unable to pass the BBB in models (as well as in vivo) [32]. Consequently, phoenixin-14 alone did not cross this BBB model in vitro.

## 4. Discussion

The BBB is crucial for regulating the transport of substances between blood circulation and the central nervous system [33]. Due to its tight barrier integrity and low permeability, the passage of molecules is strictly regulated [34]. In this study, we aimed to examine whether the novel orexigenic peptide phoenixin-14, which is secreted in the gastrointestinal tract and whose supposed receptor is mainly expressed in the hypothalamus, is able to cross the BBB to reach hypothalamic food intake-regulating regions to mediate its orexigenic effect.

To examine this gap in knowledge, an in vitro human iPSC-derived BBB was used; therefore, prior to transport studies, transendothelial electrical resistance was assessed to verify human BBB-like characteristics. The mean TEER values for phoenixin-14 were bet-ween 1325.85 and 1837.63 Ω*cm^2^. Thus, they were comparable to previously reported TEER values in this type of cell model [32]. Additionally, physiological TEER values of 1462 and 1870 Ω*cm^2^ were measured in rat and frog brain capillaries [34,35], which confirms the suitability of the iPSC-derived BBB model for transport studies.

Next, to examine the feasibility of experiments testing phoenixin-14 in the iPSC-derived BBB model, phoenixin-14′s toxicity on the hiPSC-BCECs was assessed. Here, no cytotoxic effects of phoenixin-14 were observed. Interestingly, only in comparison to treatment with 100 nMol of phoenixin-14 was the viability of cells significantly decreased after 24-h long incubation of hiPSC-BCECs with 1% SDS. In another in vitro study, phoenxin-14 was shown to prevent cytotoxic injury of microglia due to ischemia followed by reperfusion [36]. Additionally, it was shown in vivo that phoenixin-14 has protective effects on duodenal lesions associated with reduced inflammatory and oxidative markers and increased anti-oxidative contents [37], indicating cytoprotective properties of phoenixin-14. In line with the present observations, this indicates that phoenixin-14 could play a role in cell viability and death not only in the central nervous system, but also in the periphery. Further studies should investigate the underlying mechanisms in more detail.

Using an hiPSC-BCEC-based in vitro BBB model, we tested the capacity of two different concentrations of phoenixin-14 to cross the BBB. Phoenixin-14′s permeability coefficient, describing the rate at which a substance crosses a membrane, was observed to be 0.021 and 0.044 µm/min at a concentration of 1 and 10 nMol. Both of these PC are very low compared with the PC of, e.g., caffeine (PC 51.06 ± 9.90 µm/min), a solute known to cross the BBB in vivo easily [32]. Moreover, even compared with loratadine, a substance that passes the BBB slowly and which in our model had a PC of 5.78 ± 0.83 µm/min [32], the PC values of phoenixin in different doses are very low. Similarly, the clearance value, representing the percentage of circulating blood volume (in our model the apical EC medium) from which a substance was completely removed, of 1 nMol and 10 nMol of phoenixin-14 was 0.683 × 10^−6^ and 1.779 × 10^−6^ mL/min, respectively, and therefore very low. In comparison, caffeine was shown to have a clearance value of 11.11 × 10^−6^ mL/min in vivo in mice [38]. Altogether, these results indicate that there was no passage of phoenixin-14 at different concentrations via the hiPSC-BCEC-based BBB model. This is corroborated by the similarity of PC values of phoenixin-14 and dextran 40 kDa (0.0054 ± 0.000790 µm/min [32]), which due to its size is not able to pass the BBB in vivo via paracellular transport or diffusion [39].

Nevertheless, there are several characteristics of phoenixin indicating that it indeed has a significant role along the gut–brain axis. Its widespread expression in the gut and stimulated plasma levels after intake of food strongly indicate that phoenixin reaches the central nervous system due to an endocrine mechanism. Although the present results show that phoenixin-14 alone does not cross the in vitro BBB model, this does not exclude that phoenixin is able to cross the BBB in vivo, for example mediated by a co-transport mechanism. Nesfatin-1, an anorexigenic peptide expressed mainly in X/A-like cells of the stomach and exerting its effect on food intake via hypothalamic nuclei, was shown to be significantly co-expressed with phoenixin in the hypothalamus [15] and to potentiate phoenixin’s effect on reproductive hormone levels in the plasma [40]. Conversely, intracerebroventricularly injected phoenixin was shown to activate nesfatin-1-positive neurons, among others, in the PVN [16]. These observations point toward a close relationship between the two peptides and suggest that there could be a co-dependent co-transport of the peptides across the BBB, causing phoenixin alone to not able to reach a high PC and clearance value in our model. Further investigations testing the combination of both peptides in an in vitro BBB model as the present one should follow to investigate this hypothesis.

One could also assume based on the present findings that phoenixin is indeed not able to cross the BBB in vivo, but its peripherally secreted forms reach the hypothalamus via areas of the central nervous system that lack an impermeable barrier, namely the circumventricular organs including the area postrema, median eminence and adjacent neurohypophysis, organum vasculosum lamina terminalis, and subfornical organ [41], ensuring an endocrine food intake regulation by increased circulating phoenixin supposedly secreted from the gut. Future experiments testing whether phoenixin’s orexigenic properties rely on the circumventricular organs and are dependent on circulating levels thus should follow, for example using radiolabeled phoenixin-14.

In contrast, since the results shown in the present manuscript indicate that phoenixin-14 does not cross the BBB, one could assume that peripherally expressed phoenixin is not implicated in central food regulation. This hypothesis is supported by findings that the dose-dependent orexigenic property of phoenixin-14 was only observed after intracerebroventricular injection, while intraperitoneal injection did not alter food intake [23]. Furthermore, the highest concentration of phoenixin was observed in the hypothalamus, including areas involved in food intake regulation such as PVN, ARC, and VMH [4]. This could indicate that paracrine signaling is responsible for the mediation of phoenixin’s central orexigenic effect via the GPR173, whose mRNA is expressed in food regulatory centers such as PVN, VMH, and ARC [20]. Future studies applying selective inhibition of peripheral phoenxixin-14 as well as vagotomy are needed to corroborate this hypothesis.

In addition to the above-mentioned gaps in knowledge not addressed by the present study, the present study has several additional limitations. Firstly, here we applied an in vitro model which is characterized by a high correlation to the human in vivo situation [25]; however, no in vitro BBB model is able to imitate the in vivo situation perfectly and to accurately replicate the BBB’s complexity. Additional studies in animals and humans are thus necessary to confirm the present findings. Secondly, due to this study’s preliminary character, no reference substances were tested and PC values for caffeine, loratadine, and FITC dextran 40 kDa were extracted from our previous research using the same BBB model. Undeniably, internal validity could be affected by this study design. Thirdly, only two doses of phoenixin-14 were used in the transport assay; however, these were chosen to mimic the physiological differences between the fasting and fed states. The dose of 100 nMol was excluded from the transport assay due to its non-physiological property. And finally, the present study is only a brief report presenting limited data with a restricted research goal. The ultimate goal this study aimed to approach was to understand the mechanisms responsible for phoenixin-14′s orexigenic effect; however, as indicated above, many questions regarding phoenixin-14′s role along the gut–brain axis were not addressed, e.g., the potential interaction of phoenixin-14 with its receptor on brain endothelial cells.

Notably, phoenixin-14 is a pleotropic peptide also involved in numerous other physiological functions besides food intake regulation; thus, it could exert a therapeutical benefit also in obesity-independent disorders. In mice, centrally applied phoenixin-14 enhanced memory formation and prolonged memory retention [21]. Since this effect was also observed after hippocampal injection, an involvement of phoenixin-14 along the hippocampus-amygdala-prefrontal cortex pathway [42] can be assumed, potentially affecting synaptic plasticity; however, examinations of the underlying mechanisms responsible for this neuropeptide’s memory-enhancing effects, except for observations of GnRH receptor-dependent signaling [21], are scarce so far. Since phoenixin-14 was able to alleviate neuronal apoptosis and neuroinflammation [43] and reduced reactive oxygen species in neural cells such as microglia [36,44], its neuroprotective properties could also be claimed to be responsible for its effects on memory; however, this warrants further research. Importantly, phoenixin-14′s neuroprotective effect could also be used for therapeutic approaches to neuropsychiatric disorders [45], which should be investigated in the future. Furthermore, phoenixin-14 is also implicated in stress, which is indicated by observations of altered central phoenixin immunoreactivity [46] as well as a decrease in phoenixin plasma concentration [31] due to stress in the form of restraint. Interestingly, this decrease was accompanied by an increase in circulating cortisol [31] and corticosterone abolished the phoenixin-induced stimulation of NTS neuron excitability [9], indicating an involvement of phoenixin-14 in the hypothalamic-pituitary-adrenal axis; however, the exact mechanisms of its implication, such as its interaction with, e.g., central glucocorticoid receptor activity [47], need to be studied more in the future to identify potential targets in phoenixin-14′s signaling to alleviate stress.

The ultimate goal of the present research would be to understand how phoenixin-14 increases food intake, which could help to identify potential therapeutic applications of this peptide in body-weight-related pathologies. In regard to the pandemic of obesity, with more than 10% and 14% of adult men and women affected, respectively [48], as well as the substantial morbidity and mortality resulting from eating disorders [49], effective therapies to combat this threat are in high demand. Antagonism of the orexigenic peptide ghrelin, for example, was identified to inhibit growth hormone secretion, gastric emptying, and decrease postprandial glucose levels in humans [50], making it a promising therapeutic target in obesity. Since, in addition, market analyses estimate that a great proportion, namely, 35% of drugs, target GPCRs [51], understanding how phoenixin-14 functions, along with identifying its receptor and signaling pathways could supposedly ultimately lead to therapeutic applications.

## 5. Conclusions

In conclusion, the present study showed that phoenixin-14 at a dose of 1 and 10 nMol alone is not able to cross a human iPSC-derived BBB model. Thus, it appears improbable that peripherally secreted phoenixin-14 from the gut reaches the hypothalamic food regulating centers via the blood–brain barrier alone. If phoenixin-14′s passage via the BBB can be observed in vivo or in the presence of other solutes remains unclear and warrants further research. So far, there is more evidence pointing towards a centrally mediated orexigenic effect than a peripherally induced food increasing property of phoenixin-14 [23]. Since phoenixin-14 is a pleotropic peptide [18], its peripherally secreted portion could be involved in other actions instead. The application of selective inhibition of phoenixin-14 as well as genetic knockout models could help to better understand phoenixin-14′s physiological role and to identify potential therapeutic applications of phoenixin-14.

## Data Availability

The datasets used and/or analyzed during the current study are available from the corresponding author on reasonable request.
